# Evolution and implementation of One Health to control the dissemination of antibiotic-resistant bacteria and resistance genes: A review

**DOI:** 10.3389/fcimb.2022.1065796

**Published:** 2023-01-16

**Authors:** Nayeem Ahmad, Ronni Mol Joji, Mohammad Shahid

**Affiliations:** Department of Microbiology, Immunology, and Infectious Diseases, College of Medicine and Medical Sciences, Arabian Gulf University, Manama, Bahrain

**Keywords:** antibiotic resistance, antibiotic resistance genes, one health, organizations, environment

## Abstract

Antibiotic resistance is a serious threat to humanity and its environment. Aberrant usage of antibiotics in the human, animal, and environmental sectors, as well as the dissemination of resistant bacteria and resistance genes among these sectors and globally, are all contributing factors. In humans, antibiotics are generally used to treat infections and prevent illnesses. Antibiotic usage in food-producing animals has lately emerged as a major public health concern. These medicines are currently being utilized to prevent and treat infectious diseases and also for its growth-promoting qualities. These methods have resulted in the induction and spread of antibiotic resistant infections from animals to humans. Antibiotics can be introduced into the environment from a variety of sources, including human wastes, veterinary wastes, and livestock husbandry waste. The soil has been recognized as a reservoir of ABR genes, not only because of the presence of a wide and varied range of bacteria capable of producing natural antibiotics but also for the usage of natural manure on crop fields, which may contain ABR genes or antibiotics. Fears about the human health hazards of ABR related to environmental antibiotic residues include the possible threat of modifying the human microbiota and promoting the rise and selection of resistant bacteria, and the possible danger of generating a selection pressure on the environmental microflora resulting in environmental antibiotic resistance. Because of the connectivity of these sectors, antibiotic use, antibiotic residue persistence, and the existence of antibiotic-resistant bacteria in human-animal-environment habitats are all linked to the One Health triangle. The pillars of support including rigorous ABR surveillance among different sectors individually and in combination, and at national and international level, overcoming laboratory resource challenges, and core plan and action execution should be strictly implemented to combat and contain ABR under one health approach. Implementing One Health could help to avoid the emergence and dissemination of antibiotic resistance while also promoting a healthier One World. This review aims to emphasize antibiotic resistance and its regulatory approaches from the perspective of One Health by highlighting the interconnectedness and multi-sectoral nature of the human, animal, and environmental health or ill-health facets.

## Introduction

Antibiotic resistance (ABR) has emerged as one of the most serious public health threats of the twenty-first century, resulting from changes in bacteria that make antibiotics less effective. According to the UK Government-commissioned Review on Antimicrobial Resistance, by 2050, ABR might kill 10 million people each year (O'Neill, 2016). Aberrant use of antibiotics in human, animal, and environmental sectors, as well as the transfer of ABR bacteria and resistance genes among these sectors and around the world, are all factors that contribute to ABR. Animals are treated with antibiotics that are used to treat human bacterial illnesses. Given the importance and interdependence of human, animal, and environmental elements of antibiotic resistance, it makes sense to approach the issue from a One Health perspective (McEwen and Collignon, 2018).

**Figure 1:**
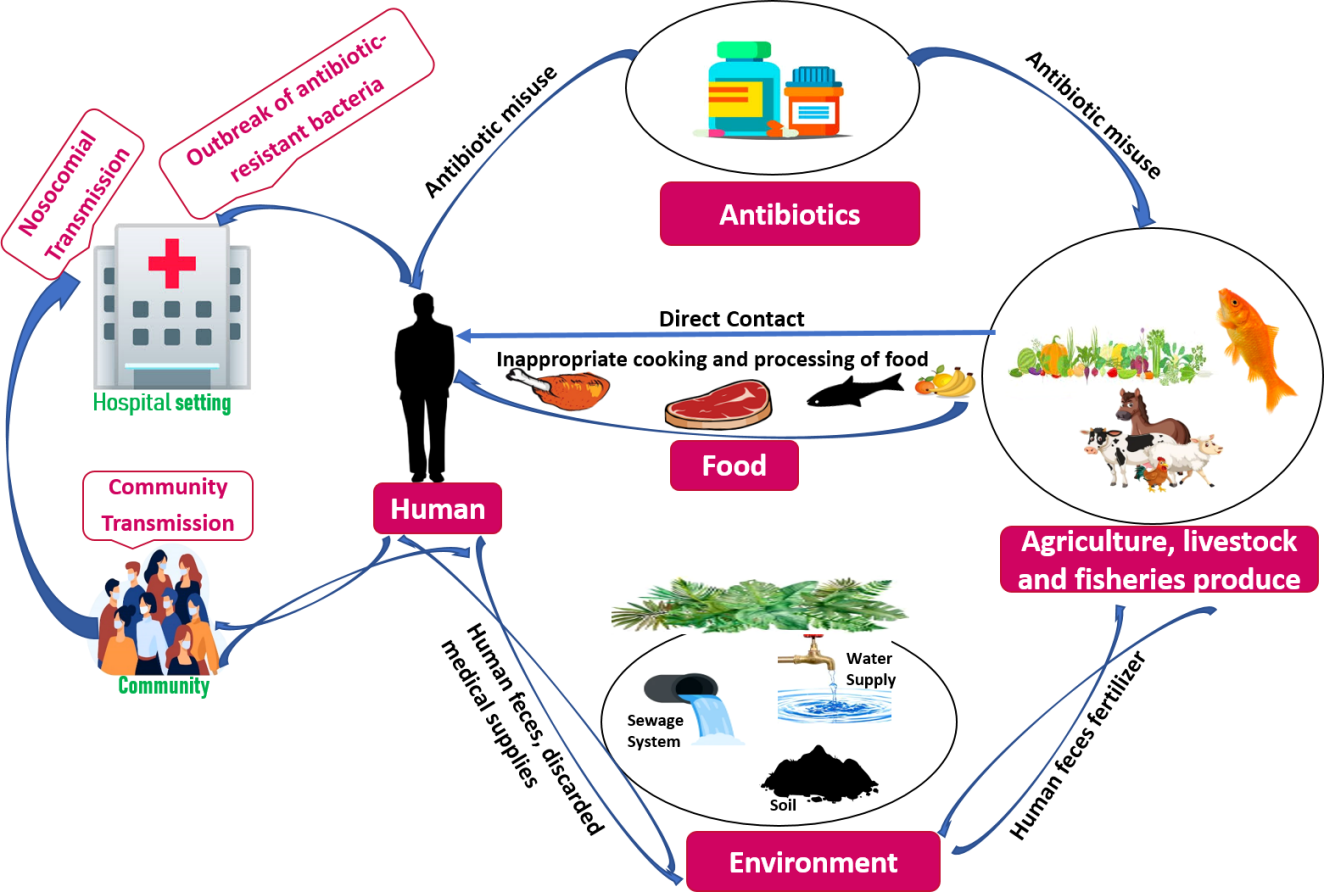
Factors contributing towards Antibiotic-Resistance (ABR) within One Health framework.

Earth’s evolution and survival are dependent on a symbiotic interaction between humans, animals, and the ecosystem in which we all live—we are all intertwined (Amuasi et al., 2020). Illnesses can originate from close interactions between humans, animals, and the environment, and their likelihood is increasing (Aarestrup et al., 2021). They make humans more susceptible to current pandemics and global health concerns like COVID-19 pandemic, antimicrobial resistance, and the rising burden of non-communicable diseases. Climate change, poverty, violence, and migration all exacerbate these global health issues (Amuasi et al., 2020). As a result, preparing for global health issues and future pandemics necessitates a special focus on comprehending the intricacies of interactions between people, animal life, and the ecosystem, as well as the various farming systems and their levels of biosafety, disease prevention and rapid outbreak containment (Aarestrup et al., 2021). One Health is a comprehensive concept that considers how human and livestock health is intertwined with the ecosystem in which they live (Aslam et al., 2021). In a global setting, the “One Health is related to One World” incorporates molecular epidemiological components that contribute to a better knowledge of ABR evolution or genetic understanding in microorganisms, human or animal hosts and the related milieu. The global spread of ABR is influenced by socioeconomic variables such as global trade, battle, dislocation, travel, and human and animal relocation (Hernando-Amado et al., 2019). In a review, Mouiche et al. stated that *Bacillus* spp., *Staphylococcus* spp., and *Vibrio cholerae* isolated from hospital devices and surfaces, water, and abattoir drain had significant multidrug resistance rates. The high ABR rates could be due to a lack of hygiene (hospital surface) and insufficient organization of expired medications. The easy spread of highly resistant bacteria from the environmental sector to humans and animals is an important factor (Mouiche et al., 2019). In agriculture, aberrant use of antibiotics has been related to an increase in ABR, because contaminating bacteria are generally treated under dosage or subjected to an ineffective drug (O'Neill, 2016; Mouiche et al., 2019). Due to the inappropriate antibiotic use in humans, communities, live stocks, and related habitats, reservoirs of resistance have evolved, resulting in resistance gene persistence in the environment (**Figure 1**).

ABR is disseminated through a variety of environmental reservoirs, including water, soil, hospitals, industrial, and agriculture waste, and numerous polluted biological niches. Pathogens containing resistance genes are more easily transported within or among individuals, live stocks, and the surrounding environment (Huijbers et al., 2015). Antibiotic-resistant bacteria may convey antibiotic resistance genes to bacteria that ordinarily reside in the human digestive system, and from there to pathogens that cause human disease. This is a possible worry concerning antibiotics usage in animal husbandry (reservoir hypothesis) (Salyers and Shoemaker, 2006). Human investigations have found some microbes including *Staphylococcus* spp., and the majority of the incorrectly used antibiotics end up in wastewater (Dickin et al., 2016). Freshwater systems are also among the natural habitats that are vulnerable to antibiotic pollution from a variety of sources, including fertilizer runoff, wastewater outflows, and seeping from nearby farms. Antibiotics in the environment, along with a larger concentration of active bacteria in freshwater, generate an environment conducive to the development of antibiotic resistance genes (Marti et al., 2014; Chen et al., 2017). As a result, freshwater ecosystems have become hubs for antibiotic resistance genes (horizontal gene transfer) and, as a result, resistance evolution. The ability of ABR bacteria and antibiotic resistance genes (ABR genes) to persist in freshwaters can raise the risk of resistant bacterial infections. Environmental survival of ABR genes can eventually revert to human and animal infections. The same freshwater body that receives wastewater also serves as a drinking water reservoir and even a recreational zone (Baquero et al., 2008). Due to the emergence and increase of ABR superbugs such as *Escherichia coli* (*E. coli*), *Klebsiella pneumoniae* (*K. pneumoniae*), and *Staphylococcus aureus* (*S. aureus*), human health is prioritized in the context of ABR, along with interdependent animal and associated environmental health (O'Neill, 2016).

It is necessary to estimate the burden of antibiotic resistance to put in place biologically, clinical, drug design, sociobehavioural, and policy measures to combat it (Essack, 2018). There are various challenges in the approaches to contain ABR. The confinement strategies centered on human, animals, or the environment alone have certain shortcomings. Some nations use integrated surveillance systems to monitor the use of ABR and antibiotics across diverse sectors. Despite these developments, evidence from published sources indicate that antibiotic consumption in both humans and animals is still rising globally and that resistant microorganisms are reemerging (Van Boeckel et al., 2015). Global human, animal, and environmental health security calls for significant actions and the execution of regulation. There are many hindrances in the way of improving antibiotic stewardship, including insufficient motivation, a lack of knowledge, improper use of antibiotics, and insufficient surveillance or legislative measures in different nations (Aslam et al., 2021).

A multi-sectoral and integrated One Health approach to addressing ABR is conceivable (Holmes et al., 2016). One Health believes that many disciplines should collaborate to ensure the worldwide health security of humans, animals, and the environment (So et al., 2015). This review emphasizes ABR and its regulatory approaches from the perspective of One Health by highlighting the interconnectedness and multi-sectoral nature of the human, animal, and environmental health or ill-health facets.

## Search strategy

We searched PubMed and Google Scholar using terms related to one health approach to combating antibiotic resistance such as “one health concept” or “antibiotic resistance mechanisms” or “antibiotic-resistant bacteria” or “antibiotic resistance genes” combined with “animals” or “poultry” or “food products” or “environment” or “water” or “human” or “one health strategies” for literature published before May 2022. One health approach/strategy was also identified through a webpage or report search. Studies of all study designs and only articles written in English were included.

Two authors (RMJ, NA) performed the initial data extraction (databases, website and report). Any discrepancies regarding study eligibility were discussed with the third author (MS) to reach a consensus. The webpages and report searches were from the official organizations sites and all the authors reached a consensus to use these websites and reports for further study. To standardize the data extraction, the following variables were collected: one health journey, descriptions, source, type of bacterial isolates, continent, country, prevalence, and type of antibiotic-resistant genes. Extracted data were entered into Microsoft Excel Sheet for further study analysis.

## Journey of “One Health” concept

In a brief, Hippocrates was the first to understand the relevance of environmental variables in human health, pushing the idea that public health was reliant on a clean environment. The relation between animal and human medication was discovered by Rudolf Virchow and William Osler, who developed the term “zoonosis” and called for veterinary medical education. In 1947, James Steele established the Veterinary Public Health Division at the Centers for Disease Control and Prevention (CDC) in Atlanta, and he made substantial contributions to the understanding of zoonotic disease epidemiology (Centre for Disease Control and Prevention).

The original One Medicine formula, created by Calvin Schwabe (veterinary epidemiologist), is where the One Health notion gets its start. It is tied to the zoonosis idea, which was coined in the 19th century by German veterinarian Rudolf Virchow. In light of these issues, Schwabe coined the term “One Medicine” to describe a need for a united approach to zoonoses from both human and animal medicine (Schwabe, 1964, 1984). **Table 1** highlights the stages of concept formation, focusing on the evolution of the formulae under which it circulates and the evolution of the One Health definition. The One Health journey demonstrates the circulation of numerous formulations that have taken up and redefined the notion. The evolution of the One Health concept uncovers the change from its conception in the initial stage to an international governance mechanism for epidemics, pandemics, and epizootics has been fueled by certain incidents that have contributed to global health emergencies, disclosing the ties between human health, animal health, the nature, and food supply in a sense of advancing global warming. The identification of these connections by health players in the relevant sectors, as well as the adoption of the One Health notion, has resulted in two developments in the concept’s evolution (Badau, 2021).

**Table 1:** Formulas and descriptions for the One Health journey*.

Date	Formula	Description	Proceedings and reasons	References
1984	One Medicine	Unification of human and animal health is being called for.	First zoonotic disease	
2004	One World, One Health	Human, animal and the environment health	Severe acute respiratory syndrome (SARS), West Nile Virus, mad cow disease, Global warming	(Wildlife Conservation Society, 2004)
2006	One World, One Medicine, One Health	Human, animal and the environment health	West Nile Virus, bioterrorism (anthrax)	(Kahn, 2006)
2007–2009	One Health Commission	Human, animal and the environment health	–	(American Veterinary Medical Association, 2008)
2008	One World, One Health	Collaborations between the three areas on a local, national, and worldwide level	H5N1	(FAO et al., 2008)
2010	One Health	Human, animal and the environment health, food standards	Following the globalization of health concerns, an integrated health approach is needed.	(FAO et al., 2010)
2011	One Health	Human and animal health, the environment, the food industry, and economic and social factors	Infectious agent circulation is increasing, increasing the possibility of pandemics (avian flu, H1N1 flu, SARS)	(Ministère des Affaires Etrangères et Européennes, 2011)

*modified from (Badau, 2021).

Since 2016, November 3^rd^ has been designated as One Health Day, to increase awareness of the holistic nature and importance of a multidisciplinary approach to solving global health issues. The four organizations: The Food and Agriculture Organization of the United Nations (FAO), the United Nations Environment Programme (UNEP), the World Organization for Animal Health (OIE), and the World Health Organization (WHO) are collaborating to bring One Health into the mainstream so that they may better prevent, anticipate, detect, and respond to global health concerns while also promoting sustainable development. The One Health High-Level Expert Panel (OHHLEP) defines One Health as an integrated, unified strategy that attempts to sustainably balance and optimize the health of people, animals, and ecosystems. It acknowledges that the human, domestic and wild animals’ health, plants, and the larger environment are all intertwined. Multiple sectors, disciplines, and communities at various levels of society are prepared to work together to promote well-being and address dangers to health and ecosystems, all while addressing the collective need for clean water, air, and energy, safe and nutritious food, combating climate change, and contributing to sustainable progressed (World Health Organisation, 2021).

All these forums affirmed that ABR needs coordinated and interdisciplinary efforts because different ecosystems participate in the acquisition, emergence, and distribution of ABR (Hernando-Amado et al., 2019).

## ABR recognized as One Health issue

The concern of antibiotic resistance was discussed for the fourth time at the United Nations General Assembly’s 71st session in 2016 (Andremont et al., 2014). When an issue becomes a public concern, it goes through steps including definition and desire for a solution, as well as political recognition, which allows for the development and implementation of public policies (Badau, 2021). The conference ended with a declaration of worldwide political commitment, demonstrating political consciousness and acknowledgment of the associations amongst the different fields engaged in antibiotic resistance, as well as its economic and societal ramifications. This commitment by global leaders highlights the importance of multi-sectoral collaboration and the implementation of national strategies per the goals of the Global Plan, which was launched in 2015 and is based on the One Health concept, which identifies the interconnectedness of human, animal, and environmental health (OMS, 2015).

The first notable publication that highlighted the necessity for a One Health strategy for antibiotic resistance was in 2014 when a report on microbial resistance surveillance was published by WHO (OMS, 2014). A description of the One Health concept is included in the paper, which includes human health, animal health and food safety. Although the description is not new, its implementation of antibiotic resistance demonstrates the onset of a phase of issue re-qualification, as evidenced by health decision-makers acknowledgment of the interconnections between antibiotic use in human health and livestock breeding, as well as the propagation of resistant strains through the food system (Badau, 2021). This is described in the following way in the report: ‘*A formal tripartite alliance is formed by WHO, OIE and FAO to improve global synchronization and encourage intersectoral collaboration amongst the public and animal health sectors, as well as food safety. Antimicrobial* *resistance (AMR) has been highlighted as one of the three priority topics for coordinated action by the FAO/OIE/WHO Tripartite’.* Multiple activities spearheaded by European and international health leaders followed this first report. Following the WHO publications, the OIE issued initiatives and resolutions emphasizing the importance of “prudent usage” of antimicrobials. The crucial point to note here is that, while their discourses emphasize international collaboration when examining each agency’s characterization of the problem, disparities emerge, implying competition for ownership of the problem, which in turn affects the description of solutions (Badau, 2021).

## Antibiotic use within the One Health pillars

Antibiotic use, antibiotic residue persistence, and the presence of ABR bacteria in the human-animal-environment habitats are all related to the One Health triangle because of the interconnection of these components in the food supply chain and ecosystem (Aslam et al., 2021). Antibiotic usage in food-producing animals has lately emerged as a major public health concern. These medicines are currently being utilized to prevent and treat infectious diseases in farm animals maintained using intense husbandry techniques (Johnston, 1998). They are also commonly given to animal feed at subtherapeutic levels for their growth-promoting qualities. These methods, on the other hand, have several negative consequences, including the induction of antibiotic resistance in bacteria and the possible spread of resistant diseases from animals to humans. Furthermore, medication residues in animal products could constitute a health concern to the general public (Al-Mustafa Zaki and Al-Ghamdi Mastour, 2002).

Rising earnings in low- and middle-income nations have fueled an unparalleled increase in animal protein demand, and the worldwide biomass of food-producing animals now exceeds that of people (Tilman et al., 2011). Between 1960 and 2013, daily consumption of animal protein in Asia raised from 7 to 25 grams per capita per day, whereas rice and wheat consumption dropped, mainly among higher-income people (Guo et al., 2000). BRICS (Brazil, Russia, India, China, and South Africa) have transitioned toward extremely cost-effective and vertically integrated intensive livestock production methods to supply this demand. Because antibiotics are required to keep the health of animals and maintain output in these production systems, rising affluence in transitioning nations is effectively promoting an upsurge in drugs and thus antibiotic resistance. Meanwhile, ABR bacteria have been found in livestock in BRICS nations and elsewhere in the developing world, where antibiotic usage for growth promotion is mainly unregulated (Van Boeckel et al., 2015). Treatment of food-producing animals with antibiotics has a global influence. Antibiotic intake in food-producing animals was assessed at approximately 63,151 tons in 2010 and is expected to increase by 67% by 2030. The growing number of animals reared for food supply accounts for two-thirds (66%) of the global increase (67%) in antimicrobial usage. The remaining third (34%) can be attributed to changes in farming practices, with a bigger proportion of animals expected to be reared in intensive farming systems by 2030 (Van Boeckel et al., 2015).

Antibiotics are mostly used in humans to treat infections and for prophylaxis. In veterinary settings, however, the use of antibiotics in pets and food-producing animals differs. Antibiotic prescriptions in pets are generally analogous to those in humans (Gouvêa et al., 2015). Antibiotics may provide 1% to 10% economic benefits when employed as poultry growth promoters, according to various research. Rather than boosting feed efficiency or production benefits, these advantages usually come from medication prophylaxis. As a result, a few significant poultry manufacturing businesses are currently promoting chicken or chicken products that have never been exposed to antibiotics. From the hatchery to the farms, to be precise (Collignon and McEwen, 2019; Hedman et al., 2020). The Food and Drug Administration (FDA) has permitted 18 antibacterial classes for use in food-producing animals. Amoxicillin, tetracyclines, colistin, neomycin, bacitracin, and lincomycin are among the antimicrobials used in animal agriculture that are crucial for human treatment, according to the World Health Organization (WHO). Of the FDA classes, penicillins, macrolides, and fluoroquinolones are the most extensively used antibiotics in human medicine, whereas penicillins, sulfonamides, and tetracyclines were the most popular among live stocks and poultry (Zalewska et al., 2021). Even though antibiotics and ABR genes are old and naturally occurring compounds, they are emerging threats linked to human-impacted habitats (Zhang et al., 2019). The recent discovery of a plasmid-mediated colistin resistance gene (mcr-1) in commensal *Escherichia coli* from pigs, pork products, and humans in China has heightened the global discussion about the magnitude of the threat posed by antimicrobial usage in livestock (Liu et al., 2016a). Antibiotic use in food-producing animals has already been reduced in several nations. Antibiotics for growth promotion, have been prohibited in the European Union since 2006. The document “Council findings on the next actions under a One Health approach to address AMR” explains the present restrictions on antibiotic usage in livestock (2016) (Zalewska et al., 2021). Walia et al. also provide an excellent overview of current global policies on antibiotic usage in livestock (Walia et al., 2019). Consumer demand is also increasing for meat free of antibiotics, with some large food vendors applying “antibiotic-free” policies for their meat traders. Improved hygiene, vaccination, and improvements in animal husbandry practices are all substitutes for using antibiotics for animal illness prevention (World Health Organisation, 2017).

When antibiotics are used indiscriminately and abusively in the environment, it results in antibiotic pollution. Antibiotics can be introduced into the environment from a variety of sources, including human wastes, veterinary wastes, and livestock husbandry waste (Gillings, 2013). Prophylactic or therapeutic antibiotics in humans pollute human waste streams, much as antibiotics used in animals for growth promotion, prevention, and treatment does. As a result, these are regarded as major sources of antibiotic contamination in the environment (Dolliver and Gupta, 2008). Nonetheless, both antibiotics and resistance genes will be present in these waste streams; both are pollutants, and their fate in the environment will be different (Martinez, 2009). Antibiotics can enter the environment by soil supplementation with animal dung, irrigation with farm wastewater, or inadvertent discharge from agricultural runoffs (Spiehs and Goyal, 2007). The soil has been recognized as a reservoir of ABR genes, not only because of the presence of a wide and varied range of bacteria capable of producing natural antibiotics (Lang et al., 2010) but also for the usage of natural manure on crop fields, which may contain ABR genes or antibiotics: only a limited fraction of antibiotics are absorbed or metabolized by animals, with approximately 75% of the administered dose being emitted in the urine or feces (Zhang and Zhang, 2011). Since the 1950s, antibiotics have been employed to control bacterial infections in high-value fruit, vegetable, and ornamental plants (McManus et al., 2002). Tetracyclines, β-lactams, lincosamides, aminoglycosides, pleuromutilins, macrolides, and sulphonamides are some of the more extensively used medications in agriculture around the world, and they are causing increasing scientific worry about their potential side effects and risk management actions (Baynes et al., 2016). Mostly oxytetracycline and streptomycin are often used as antibiotics on plants today. Although oxytetracycline resistance in plant pathogens is rare, the introduction of streptomycin-resistant *Erwinia amylovora*, *Pseudomonas* spp., and *Xanthomonas campestris* strains has hindered the therapeutic action of several plant diseases (McManus et al., 2002). Many researchers have found ABR pathogens or different types of ABR genes in fruits and vegetables before harvest *via* soil and fertilizers such as irrigation water, manure, and sewage sludge. This is a severe concern since any contaminated raw veggies could act as vehicles for the spread of ABR genes or antibiotic-resistant bacteria to people (Hölzel et al., 2018). Antibiotics in plant tissues at sub-inhibitory concentrations have been discovered to be possible drivers of antibiotic resistance in endophytic bacteria, and modest quantities of antibiotics, such as tetracycline, can cause horizontal gene transfer (HGT) between bacteria (Redondo-Salvo et al., 2020).

## Antibiotic resistance mechanisms in bacteria

**Figure 2:**
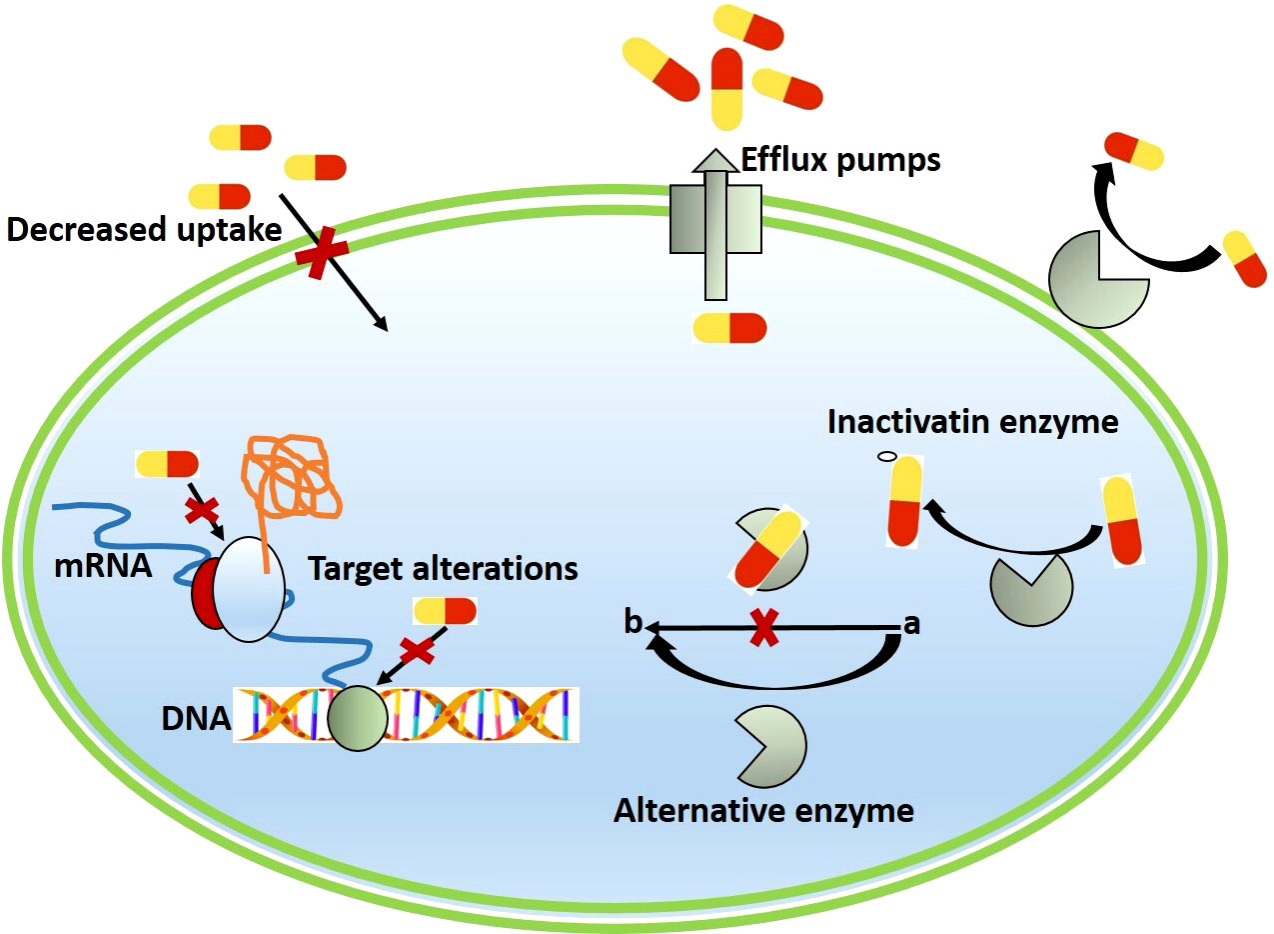
Schematic representation of different mechanisms of antimicrobial resistance in bacterial antibiotic Inactivation.

Bacteria develop resistance to antibiotics through multiple mechanisms, which are explained in detail (**Figure 2**).

### Inactivation of antibiotic

Several bacterial pathogens have developed resistance to β-lactam antibiotics by adapting the antibiotics or releasing enzymes that inhibit or degrade the chemical properties of antibiotics (Wright, 2005). Bacteria inactivate antibiotics in one of two ways: by degrading the drug or by transferring a chemical group to it. β-lactamases are a wide group of enzymes that hydrolyze β-lactams. Another antibiotic that can be blocked by hydrolysis *via* the tetX gene is tetracycline (Kumar et al., 2013; Blair et al., 2015). Plasmids are mobile genetic materials found in bacteria that contain genes that code for resistance to specific antibiotics. Most antibiotics with a β-lactam ring, such as penicillin, amoxicillin, ampicillin, piperacillin, ceftazidime, imipenem, and others, are becoming dysfunctional due to the production of the β-lactamase enzyme, which is causing hydrolysis of the amide bond in the β-lactam ring (Wilke et al., 2005). Transfer of a chemical group to the drug is perhaps the most common method of drug inactivation. Transfer of acetyl, phosphoryl, and adenyl groups is most commonly used. Acetylation is now the most widely used pathway, and it is effective against chloramphenicol, aminoglycosides, fluoroquinolones, and streptogramins, Phosphorylation and adenylation be effective against aminoglycosides (Schwarz et al., 2004; Robicsek et al., 2006; Ramirez and Tolmasky, 2010; Blair et al., 2015). So far, more than 1000 β-lactamases have been identified (Bush and Fisher, 2011).

### Alteration of drug target

Antimicrobial agents bind to a specific site and alter normal activity; this is referred to as the target site. The alteration of these target sites causes bacteria resistant to some antibiotics. The alteration or modification of the target site could be caused by bacteria-produced constitutive and inducible enzymes. *Streptococcus* spp. resist the action of macrolides, streptogramin B and lincosamides, by preventing them from attaching to the ribosomal subunit (50S) and limiting protein synthesis *via* post-transcriptional alteration of the 23S rRNA component of this ribosomal subunit (Kataja et al., 1998). Antibiotics may target a variety of other components in the bacterial cell, and the bacteria may change those targets to enable drug resistance. One method of β-lactam resistance, which are virtually exclusively employed by gram-positive bacteria, is a change in the structure or number of PBPs (penicillin-binding proteins). PBPs are transpeptidases that help the cell wall to synthesize peptidoglycan. The drug concentration that can bind to that target is affected by the number of PBPs. A structural alteration (mecA gene acquisition in *S. aureus*) may diminish or prevent drug resistance (Reygaert, 2009; Beceiro et al., 2013). Vancomycin is a glycopeptide that acts by attaching to D-Ala-D-Ala and producing a cap, causing the polypeptide chain to lose cross-linking. By altering the D-Ala-D-Ala binding site at the C-terminus to D-alanyl-D-serine or D-alanyl-D-lactate, bacteria become vancomycin-resistant (Hiramatsu, 2001). Antibiotics that inhibit bacterial growth by protein synthesis inhibition or transcription include aminoglycosides, chloramphenicol, tetracyclines, streptogramin, oxazolidinones, fusidic acid, and macrolides. Bacterial resistance to these antibiotics is caused by the mechanism of specific target modification (Happi et al., 2005).

### Efflux mechanism

In bacteria, intrinsic resistance is caused by efflux pump proteins (Lomovskaya and Bostian, 2006).09265851281 There is evidence in the literature that the resistance mechanism to almost all antibiotics is active efflux (Lin et al., 2002). The bulk of bacterial efflux pumps is non-drug specific proteins that recognize and eject chemicals, antibiotics, and structurally distinct compounds without changing or degrading the drug (Kumar and Schweizer, 2005). Bacteria have chromosomally determined efflux pump genes. A few are constitutively expressed, whereas others are overexpressed or induced in retort to environmental signals or the existence of a particular surface. The major function of efflux pumps is to remove hazardous molecules from the bacteria, and many of these pumps can transport a wide range of compounds. The resistance capabilities of most of these pumps are controlled by the carbon source available (Villagra et al., 2012; Blair et al., 2014).

Antibiotics or chemical compounds are expelled from the cell, resulting in a low concentration of antibiotics that do not affect the bacteria. Gram-negative bacteria have a double outer membrane with a phospholipid (inner layer) and a lipopolysaccharide lipid A moiety (outer layer), according to their formation. Because of the content of the outer membrane, drug absorption and transport throughout the outer membrane of gram-negative bacteria are relatively difficult, and drug transport is facilitated by water-filled channels formed by porin proteins. Antimicrobial molecules can enter the bacterial cell through the outer membrane *via* porin or through the lipid bilayer. One of the most powerful predictors of the entry mode is the chemical composition of the drug molecule. Chloramphenicol and fluoroquinolones, for example, penetrate Gram-negative bacteria also with the assistance of porin (Nikaido, 2003).

### Limitation of drug uptake

Bacteria, due to their structure and morphology, can be intrinsically resistant to these antibiotics. In gram-negative bacteria, for example, lipopolysaccharide (LPS) acts as a physical barrier that protects large molecular groups (Maldonado et al., 2016). Drugs are internalized by these bacteria *via* porin channels, which permit the uptake of hydrophilic compounds in general. The two main mechanisms of antimicrobial resistance are mutations that change their selectivity or reduce the number of expressed porins (Blair et al., 2014; Choi and Lee, 2019). Gram-negative bacteria, which lack an outer membrane, have a peptidoglycan cell wall, and drug uptake is not as restricted. Pathogenic bacterial species, such as *Staphylococcus aureus*, have designed the system to limit the number of drugs that enters the cell by thickening the cell wall (Cui et al., 2003; García et al., 2017). Porin channels are frequently used by substances to enter the cell. In general, gram-negative bacteria porin channels offer access to hydrophilic compounds (Gill et al., 1998; Blair et al., 2014). Porin mutations and alterations in the selectivity of the porin channel can both reduce drug absorption (Kumar and Schweizer, 2005). As the number of porins diminish, Enterobacteriaceae members are known to become resistant. As a mechanism for carbapenem resistance, these bacteria diminish porin quantities (Chow and Shlaes, 1991; Cornaglia et al., 1996). The biofilm formation by a bacterium is another well-known phenomenon in bacterial colonization. These biofilms might cover a dominant organism (as seen in the lung by *Pseudomonas aeruginosa*) or a diverse community of bacteria, as seen in the gut biofilm community. For harmful microorganisms, biofilm protects the bacteria from host immune system attack as well as antimicrobial agents. The biofilm matrix’s thick, sticky comparability, which contains proteins, polysaccharides, and DNA from the resident bacteria, makes it harder for antibiotics to reach the bacteria. As a result, much-increased levels of the drugs are required for effectiveness. Furthermore, because the bacterial cells are sessile in the biofilm, antibiotics that target growth, and cell division have little effect. A significant reflection of biofilms is that the proximity of the bacterial cells likely facilitates horizontal gene transfer. This means that these bacterial communities may find it easier to share antimicrobial resistance genes (Mah, 2012; Soto, 2013; Van Acker et al., 2014).

Antimicrobial resistance can be either acquired or intrinsic. Intrinsic antimicrobial resistance is a bacterial species or genus’ inherent resistance to a certain antibiotic. As a result, treating with this specific antibiotic is unlikely to be effective (Aarestrup, 2005). It may further exacerbate a medical problem by causing sub-infection with other intrinsically resistant bacteria, such as *Clostridium difficile* in humans (Viseur et al., 2011) or *Trueperella (Arcanobacterium) pyogenes* in cattle (Catry et al., 2008). Antimicrobial resistance develops when a sensitive strain evolves to become resistant as a result of recent mutations. This can occur as a result of either a spontaneous mutation within a microbial community or the acquiring of a specific resistance gene through HGT. Conjugation, transformation, and transduction are the three primary mechanisms of HGT between bacteria. These can be found in natural sources including soil, and water, in humans and animals’ digestive systems, and food. Conjugation is the mechanism of DNA transfer between the living donor and recipient bacterial cells (Verraes et al., 2013). The process of transformation occurs when environment bare DNA is taken up by bacteria (Kelly et al., 2009). The bacteriophage-mediated transfer process is known as transduction. Because bacteriophages have a limited host range, transduction between closely related bacterial strains happens in general. A phage’s transducing ability is not restricted to bacteria that can be infected but can be far broader (Holmfeldt et al., 2007). The maintenance of the ABR gene in bacterial populations and multidrug resistance development are all aided by genetic elements like integrons, plasmids, and transposons (Revilla et al., 2008; Ronni Mol et al., 2022; Singh et al., 2022). Other less well-known DNA transfer methods may occur in nature. These include translocation (vesicle-mediated) from one cell fused with another (Gram-negative bacteria), ABR gene transfer by virus-like particles, and cellular fusion causing entire genome mixing to occur in bacteria (Verraes et al., 2013).

## Distribution of ABR bacteria and ABR genes at the human, animal and environment interface

ABR is a phenomenon that emerges at the intersection of a complex One Health system; human health is assumed to be influenced by a variety of factors, including industrial, agricultural, and veterinary activities, as well as environmental factors (Hernando-Amado et al., 2019). These various drivers can be divided into two parts in the context of ABR: “selection,” mostly caused by the use of antibiotics, and “transmission” of resistant organisms between each connected sector along the axis of humans, animals, and environments. Although the magnitudes of these effects are poorly characterized and likely to be antibacterial, resistance mechanism, and organism specific, it is generally known that antibiotic use in animals cause ABR (Holmes et al., 2016). The majority of the world’s antibiotic use (73%) is found in animals reared for food (Van Boeckel et al., 2019)

Antibiotic usage in animals can have both direct and indirect health consequences for humans (Landers et al., 2012): Some of the direct effects could be;

- The chance of resistance colonization or infection in humans is enhanced when farm animals are given antibiotics.
- Antibiotic-resistant bacteria-infected food, causing an outbreak of resistant diarrheal illness.
- Antibiotic-affected meat product consumption can cause resistance in the normal microbiota of the human gastrointestinal tract.

Some of the indirect effects could be;

- Animal transportation causes resistant bacteria to spread throughout the route.
- Antibiotic-resistant bacteria in animals have mobile genetic components that are integrated into pathogens that cause human illness.
- Water contamination and changes in human flora are caused by resistant bacteria from animal waste used as fertilizer.

Animal or human colonization and illness occur when companion animals come into touch with antibiotic-resistant pet food (Landers et al., 2012). *Acinetobacter baumannii, Pseudomonas aeruginosa*, and *Enterobacteriaceae* are the top priority microorganisms for ABR prevention, according to the WHO. *Salmonella* spp. and *Campylobacter* spp., both of which are susceptible to 3^rd^ generation Cephalosporins and fluoroquinolones, are other bacteria that can cause foodborne illness transmission. The WHO considers these to be high-priority rather than critical (World Health Organization, 2017). Of the *Enterobacteriaceae*, *E. coli* has the highest risk of animal-human spread and is a significant ABR bacterium, carrying various genes for antibiotic resistance. *E. coli* is a commensal that colonizes human and animal guts and is found in soil, water, plants, and vegetables. Human gastroenteritis, urinary tract infection and septicemia are all caused by pathogenic *E. coli* strains (Van den Bogaard and Stobberingh, 2000). Very few studies have examined the resistance profiles of ABR bacteria and ABR genes in food-producing animals and directly-exposed humans. (Donkor et al., 2012) assessed MDR *E. coli* in cattle and their farmers in Ghana. Animal and human *E. coli* isolates showed high levels of MDR ABR (70.6% and 97.7%, respectively), although animal-derived isolates had high resistance to five antimicrobials (cefuroxime, co-trimoxazole, tetracycline, ampicillin and amikacin) and human-derived isolates had higher resistance to chloramphenicol and gentamycin. Dhaka et al. (2016) studied the presence of resistant bacteria and genes in all three domains of humans, animals and the environment. They assessed ABR in diarrhoeagenic *E. coli* (DEC) in animals, food products, environmental samples and infants with diarrhoea in India. Of the four DEC pathogens, enteroaggregative *E. coli* was the most common with a prevalence of 16.5% in infants, 17.9% in young animals, 16.2% in foods and 3.4% from environmental sources. Around 86% of isolates were resistant to three or more classes of antibiotics. Studies comparing the profiles of resistance in humans and animals have mainly relied on indirect connections. Both community-acquired *E. coli* infections and colonized healthy persons in China were showing an increase in the ESBL-producing enzyme CTX-M-55. Prior to that, the enzyme was primarily found in food-producing animal creatures (globally since 2002), suggesting a potential animal to human transmission (Rousham et al., 2018). The prevalence of some of the ABR bacteria from food and food products and the environment is shown in **Table 2**.

**Table 2:** Some studies reporting the prevalence of ABR bacteria isolated from food producing animals, food products and environment* [modified from Hosain et al, 2021 [108].

Country	Specimens	ABR bacteria	Prevalence (%)	References
ASIA				
China	Milk	*Salmonella *spp.	29.4	(Cao et al., 2021)
	Vegetables	*E. coli*	13.8	(Mei et al., 2021)
	Milk	*Salmonella *spp.	1.3	(Lan et al., 2017)
	Chicken	*E. coli*	73.9	(Li et al., 2021)
	Pork	*E. coli*	42.9	
	Pork	*E. coli*	59.6	(Li et al., 2020)
	Mutton	*E. coli*	52.6	
	Milk	*E. coli*	52.4	
	Duck	*E. coli*	36.4	
	Beef	*E. coli*	35.3	
	Chicken	*E. coli*	33.3	
	Ready to eat foods	*E. coli*	12.9	
India	Bovine	*E. coli*	71.43	(Manna et al., 2006)
	Poultry	*E. coli*	63.2	(Sivagami et al., 2020)
	Piglets	*E. coli*	80	(Dutta et al., 2011)
	Bovine	Shiga toxin producing* E. coli*	17	(Arya et al., 2008)
	Poultry	*Salmonella *spp.	100	(Samanta et al., 2014)
	Beef	Enterohaemorrhagic *E. coli*	12.5	
	Goat meat	Enterohaemorrhagic *E. coli*	5.7	
	Buffalo meat	Enterohaemorrhagic *E. coli*	13	(Sethulekshmi et al., 2016)
	Water samples	*E. coli*	21.1	(Shahid et al., 2012)
		*Klebsiella* spp.	4.4	
		*Citrobacter* spp.	7.7	
		*Pseudomonas* spp.	10	
		*Acinetobacter* spp.	4.4	
Pakistan	Meat	*E. coli*	75	(Malik and Memona, 2010)
Bhutan	Pig fecal sample	*E. coli*	2.4	(Hosain et al., 2021)
Iran	Milk	*E. coli*	78	(Hassani et al., 2022)
		*L. monocytogenes*	47	
		*S. aureus*	25	
		*Salmonella *spp.	21	
	Kebab	*E. coli*	70	(Rajaei et al., 2021)
		*Salmonella *spp.	58	
		*L. monocytogenes*	50	
		*S. aureus*	36	
	Hamburger	*E. coli*	48	
		*Salmonella *spp.	10	
		*L. monocytogenes*	22	
		*S. aureus*	22	
		*P. aeruginosa*	6.5	
		*Acinetobacter* spp.	2.2	
		*S. aureus*	4	
		*Enterococcus faecalis*	17.1	
		*Enterococcus faecium*	6	
Indonesia	Poultry	*E. faecalis*	84.5	(Usui et al., 2014)
Lebanon	Lettuce	*E. coli*	42.3	(Halablab et al., 2011)
	Parsley	*E. coli*	13.8	
	Lettuce	*S. aureus*	51.5	
	Parsley	*S. aureus*	38	
Myanmar	Poultry	*Salmonella *spp.	52.2	(Moe et al., 2017)
Nepal	Buffalo and poultry meat	*E. coli*	52.5	(Saud et al., 2019)
		*Proteus *spp.	77.7	
		*S. aureus*	40.0	
Sri-Lanka	Cattle, poultry and pig	*S. aureus*	65	(Jayaweera and Kumbukgolla, 2017)
South Korea	Cattles, pigs, chickens	Extended-spectrum cephalosporins resistant *Salmonella virchow*	63.8	(Na et al., 2020)
Thailand	Poultry	ESBL-producing *Salmonella typhimurium*	77.3	(Padungtod et al., 2008)
	Pig	ESBL-producing *Salmonella typhimurium*	40.4	
	Pig	ESBL-producing *E. coli*	77	(Boonyasiri et al., 2014)
	Pork	ESBL-producing *E. coli*	61	
	Pork	*A. baumannii and P. aeruginosa*	40	
	Poultry	ESBL-producing *E. coli*	40	
	Poultry meat	ESBL-producing *E. coli*	50	
Malaysia	Meat products	*L. monocytogenes*	8.57	(Marian et al., 2012)
Saudi Arabia	Raw meat	*E. coli*	4.2	(Aabed et al., 2021)
	Fresh vegetables	*E. coli*	4.2	
	Water	*E. coli*	4.2	
	Air	*E. coli*	4.2	
	Beef meat	*E. coli*	22.22	(Hemeg, 2018)
Kuwait	Chicken	*E. coli*	80	(Eltai et al., 2020)
AFRICA				
South Africa	Producer distributor bulk milk	Diarrheagenic *E. coli*	57.5	(Aijuka et al., 2018)
	Irrigation water	Diarrheagenic *E. coli*	23.4	
	Irrigated lettuce	Diarrheagenic *E. coli*	14.14	
	street vendor coleslaw	Diarrheagenic *E. coli*	4.8	
	Raw intact meat, raw processed meat, and ready-to-eat meat from cattle, game, sheep, pork, and poultry	*E. coli* (domestic market)	1.59	(Madoroba et al., 2022)
		*E. coli* (ports of entry)	1.54	
Ghana	Chicken meat	*E. coli*	81.3	(Rasmussen et al., 2015)
	Poultry meat	*E. coli*	23	(Eibach et al., 2018)
		*K. pneumoniae*	17.5	
Nigeria	Wastewater Treatment Plant effluent	*E. coli*	21.8	(Chigor et al., 2022)
	Tap water	*E. coli*	5.3	
	Vegetables from greenhouse	*E. coli*	24.4	
	Vegetables from farms	*E. coli*	29.2	
	Vegetables from markets	*E. coli*	19.14	
	Cattle	ESBL-producing *E. coli*	45.4	(Aworh et al., 2022)
	Abattoir environment	ESBL-producing *E. coli*	13.4	
Tunisia	food-producing animals (sheep, cattle, chickens, rabbits, horses, and dromedaries)	cefotaxime-resistant *E. coli*	13.8	(Ben Sallem et al., 2012)
Algeria	Ready-to-eat sandwiches	*E. coli*	85.7	(Yaici et al., 2017)
		*K. pneumoniae*	52.3	
		*K. oxytoca*	4.7	
NORTH AMERICA				
Mexico	Irrigation water	*E. coli*	43.6	(Canizalez-Roman et al., 2019)
Alberta	Retail ground beef and Beef Processing Plant	*Enterococcus faecium*	1.3	(Holman et al., 2021)
		*E. faecalis*	18.18	
Virginia	Water from agricultural ponds	*Salmonella* spp.	19	(Truitt et al., 2018)
Canada	Imported plant-based food products	*E. coli*	9.1	(Jung and Rubin, 2020)
		*Salmonella* spp	1.4	
		ESBL producing *Enterobacter* spp.	1.4	
		*K. pneumoniae*	1.4	
		*S. aureus*	4.9	
		*Enterococcus* spp.	46.2	
New York	Surface water	*E. coli*	33	(Jones et al., 2014)
		*Salmonella* spp.	43	
	Produce fields	*L. monocytogenes*	15	(Strawn et al., 2013)
		*Salmonella* spp.	4.6	
		Shiga toxin-producing *E. coli*	2.7	
Georgia	Irrigation water	*Salmonella* spp.	42.8	(Lee et al., 2018)
SOUTH AMERICA				
Brazil	Cheese	*E. coli*	71.4	(Dos Santos et al., 2022)
	Fish (Arapirama gigas)	quinolone-resistant *E. coli*	85	(Lima et al., 2022)
	Irrigation water	*E. coli*	84.8	(Decol et al., 2017)
	Lettuce	*E. coli*	38.3	
	Organic vegetables	*E. coli*	41.5	(Maffei et al., 2013)
	Conventional vegetables	*E. coli*	40	
Ecuador	Broiler farms	Third-generation cephalosporin-resistant *E. coli*	91.7	(Ortega-Paredes et al., 2020)
	broiler carcasses	Third-generation cephalosporin-resistant *E. coli*	77	(Ortega-Paredes et al., 2020)
	Poultry farms	*S. Infantis*	94.4	(Sánchez-Salazar et al., 2020)
EUROPE				
United Kingdom	Beef	ESBL-producing *E. coli*	1.9	(Randall et al., 2017)
	Pork	ESBL-producing *E. coli*	2.5	
	Chicken	ESBL-producing *E. coli*	65.4	
	Fruits and Vegetables	–	–	
	Chicken	ESBL and/or AmpC -producing *E. coli*	45/13.6 (2016/2018)	(Randall et al., 2021)
		ESBL- producing *E. coli*	28.8/7.4 (2016/2018)	
		AmpC- producing *E. coli*	15.3/5.2 (2016/2018)	
		AmpC+ESBL -producing *E. coli*	1/1 (2016/2018)	
Portugal	Broiler meat	Third-generation cephalosporin-resistant *E. coli*	30.3	(Clemente et al., 2021)
		Fluoroquinolone resistant *E. coli*	93.3	
Turkey	Chicken	*E. coli*	81	(Pehlivanlar Önen et al., 2015)
	Beef meat	*E. coli*	7	
Spain	dairy cattle herds	*E. coli*	32.9	(Tello et al., 2020)
	beef cattle herds	*E. coli*	9.6	
	sheep flocks	*E. coli*	7	
Poland	Fish and sea food	Methicillin-resistant *Staphylococcus aureus*	59	(Kukułowicz et al., 2021)
Belgium	Broiler farm	ESBL-producing *E. coli*	85	(De Koster et al., 2021)
		Ciprofloxacin resistant *E. coli*	88	
	Pig farms	ESBL-producing *E. coli*	37	
		Ciprofloxacin resistant *E. coli*	33	
Netherlands	Broiler farm	ESBL-producing *E. coli*	27	(De Koster et al., 2021)
		Ciprofloxacin resistant *E. coli*	82	
	Pig farms	ESBL-producing *E. coli*	4	
		Ciprofloxacin resistant *E. coli*	11	
	Imported culinary herbs	Cefotaxime resistant Enterobacteriaceae	42	(Veldman et al., 2014)
Norway	Dairy cattle farms milk filters	*Campylobacter* spp.	4	(Idland et al., 2022)
		*L. monocytogenes*	13	
		Shiga toxin-producing *E. coli*	7	
	Bulk tank milk	*Campylobacter* spp.	3	
		*L. monocytogenes*	0	
		Shiga toxin-producing *E. coli*	1	
Greece	Dairy (cattle, sheep and goat) farms	*S. aureus*	47.8	(Papadopoulos et al., 2019)
		Methicillin-resistant *Staphylococcus aureus*	4.1	
AUSTRALIA/OCEANIA				
Australia	Poultry	Polymyxin resistant Gram-negative bacteria	26.8	(Bean et al., 2020)
	River WWTP Discharge	*E. coli*	–	(Watkinson et al., 2017)
	River Population	*E. coli*	–	
	Crustaceans and environmental isolates, freshwater and marine water fishes	*Vibrio* spp.	60	(Akinbowale et al., 2006)
		*Aeromonas* spp.	21	
		*Photobacterium* spp.	4	
		*Pseudomonas* spp.	4	
		*Edwardsiella tarda*	2	
		*Hafnia alvei*	1	
		*Flavobacterium* spp.	2	
		*Plesiomonas shigelloides*	1	
		*Staphylococcus and Micrococcus* spp.	4	
		*Citrobacter* spp.	2	
New Zealand	Vegetables (organic farms)	*E. coli*	92	(Wadamori et al., 2016)
		*S. aureus*	44	
	Vegetables (traditional farms)	*E. coli*	88	
		*S. aureus*	27	

*modified from (Hosain et al., 2021).

Poultry farming poses a high risk of ABR development, especially in unregulated small-scale enterprises as seen in low-income countries. Poultry farming is extremely profitable and well-suited to areas with limited space (Shanta et al., 2017). Antibiotic use is more widespread in poultry than in other livestock, and resistance is much more likely to develop amid overcrowding and poor sanitation (Graham et al., 2017). Antibiotic usage in aquaculture is significant as a possible driver of ABR in aquatic systems (Taylor et al., 2011). In Vietnam, 72.3% of the freshwater fish and shrimp farms surveyed utilized at least one antibiotic (Pham et al., 2015). Farms having a larger density of fish or shrimp, as well as a higher overall annual production, used more antibiotics. The researchers also looked at fish products sold in local markets, but without a clear link to farms. Fluoroquinolone and tetracycline antibiotic residues were found in 26.9% of retail shrimp and fish samples from local markets, indicating a lack of sufficient withdrawal times on farms. Quinolone and ESBL resistance genes have been found in market fish farmed throughout southern China’s Guangdong (Jiang et al., 2012). Several studies have reported the presence of ABR genes in the environment (**Table 3**). At the human-animal interface, some studies have reported minimal interrelationship of MDR bacterial pathogens. Ludden et al. (2019) analyzed *E. coli* core genomes isolated from animal farms and retail meat in East England. In total, 41 distinct resistance genes were found in various quantities in livestock. ESBLs, strA, strB, sul1, sul2, tetA, and tetB are all highly common genes. They discovered genetically different isolates from cattle and humans, indicating that human illnesses associated with *E. coli* did not originate directly from livestock (Ludden et al., 2019). Animal and food transportation have also contributed to the spread of ABR worldwide. Pigs raised in China were the first to exhibit mcr-1, a plasmid-mediated resistance mechanism to the antibiotic colistin [52]. Since its identification in 2015, mcr-1 has been found in Enterobacteriaceae strains from five different continents, including those found in humans, food, farm and wild animals, and aquatic settings (Rousham et al., 2018).

**Table 3:** Some studies reporting the distribution of ABR genes among the bacterial isolates from food producing animals, and food products.

S/No.	Type of specimens	Bacteria isolated	ABR genes	References
1.	Poultry, beef, pork, milk, cheese and vegetables	*E. coli*	CTX-M-1	(Irrgang et al., 2018)
2.	Imported vegetables, fruits and spices	*E. coli*, *Salmonella spp* *ESBL producing Enterobacter spp* *K. pneumoniae* *S. aureus* *Enterococcus spp*	CTX-M-1, CTX-M-15, CTX-M-27, SHV-106, mecA, qnr, aac(6)-ib-cr	(Jung and Rubin, 2020)
3.	Bovine milk	E. coli	TEM, SHV	(Hassani et al., 2022)
		*L. monocytogenes*	–	
		*S. aureus*	mecA, blaZ	
		*Salmonella* spp.	TEM, SHV	
4.	Chicken meat, turkey meat, beef, pork, minced meat. cheese	Cefotaxime resistant *E. coli*	CTX-M, SHV, TEM	(Kaesbohrer et al., 2019)
5.	Fresh vegetables and ready to eat prepacked salads	Gram negative bacteria	SHV-12, CTX-M-1, CTX-M-15, ACC-1, DHA-1, VIM-1, IMP-1	(Iseppi et al., 2018)
6.	Kebab and hamburgers	*E. coli*	TEM, SHV	(Rajaei et al., 2021)
		*Salmonella* spp.	TEM, SHV	
		*L. monocytogenes*	mecA	
		*S. aureus*	mecA	
7.	Retail pork, pigs, broiler chickens	*Salmonella* spp.	pse-1, tetA, tetB, ant(3′′)-Ia, sul 1, sul 2	(Zishiri et al., 2016)
8.	Beef meat	*E. coli*	TEM, SHV	(Hemeg, 2018)
9.	Meat products	*E. coli*	TEM	(Hamed et al., 2017)
10.	Ground Beef	*E. coli*	CTX-M	(Rebbah et al., 2018)
11.	Food animals (cattle, pigs, poultry)	*E. coli*	CTX-M	(Zheng et al., 2012)
12.	Raw beef and beef products	*E. coli*	mcr-1, CTX-M-28	(Sabala et al., 2021)
13.	Ready to eat cheese	*E. coli*	mcr-1	(Hammad et al., 2019)
14.	Poultry	*E. coli*	mcr-1	(Lima Barbieri et al., 2017)
15.	Chicken & Pork	*E. coli*	TEM-1D, CTX-M-9, CTX-M-1, OXA-7, mcr-1, sul1, sul2, sul3	(Li et al., 2021)
16.	Vegetables, eggs, chicken, milk, meat	*E. coli*	ESBL	(Rasheed et al., 2014)
17.	Cow’s milk	*S.aureus*	blaZ, mecA	(Sadat et al., 2022)
18.	Retail meat and broiler chickens	*S.aureus*	mecA, tetK, blaZ	(Mkize et al., 2017)
19.	Milk	*Klebsiella* spp	aadA22, arr-3, aac(3)-Id, cmlA, TEM, ereA2, SHV, OXA, CTX-M	(Ahmed and Shimamoto, 2011)
20.	Vegetables (leafy Vegetables, leafy Herbs. tomatoes, green onions, cucumbers, berries)	*Enterobacteriaceae*	ESBL	(Salmanov et al., 2021)
		*S.aureus*	MRSA	
21.	Beef, pork and chicken	ESBL-producing *E. coli*	CTX-M, SHV, TEM	(Randall et al., 2017)
22.	Chicken & beef	*E. coli*	CTX-M, fosA3, fosA4, mcr-1	(Ramadan et al., 2021)
23.	Fruits	*E. coli*	CTX-M, TEM, SHV, mcr-1	(Montero et al., 2021)
	Irrigation water	*E. coli*	CTX-M, TEM, SHV, OXA, mcr-1	
	Vegetables	*E. coli*	CTX-M	
	Environmental samples (drinking water, drain, sewage water)	*E. coli*	CTX-M	(Shahid et al., 2012)
		*Klebsiella spp*	CTX-M	
		*Citrobacter spp*	CTX-M	
		*Pseudomonas spp*	CTX-M	
		*Acinetobacter spp*	CTX-M	
24.	Chicken and meat	*E. coli*	CTX-M-1, CTX-M-14, CTX-M-55, CMY-2, TEM-1, SHV-12, mcr-1	(Hassen et al., 2020)
25.	Healthy broilers	*E. coli*	CMY-2, CTX-M-3, CTX-M-15, CTX-M-27, CTX-M-14	(Mikhayel et al., 2021)
26.	Chicken	*E. coli*	mcr-1, CTX-M-55, CTX-M-65, TEM	(Nakayama et al., 2022)
27.	Chicken	*E. coli*	mcr-1, mcr-2	(Vounba et al., 2019)
28.	Chicken	*E. coli*	CTX-M-1	(Randall et al., 2021)
29.	Poultry	*E. coli*	mcr-1	(Hu et al., 2022)
30.	Meat	*E. coli*	CTX-M-1, CTX-M-15, CTX-M-32, CTX-M-55, CTX-M-65, CTX-M-27, CTX-M-9, CTX-M-14, SHV-12, TEM-52, CMY-2, qnrB, qnrS, aac (6’)-Ib-type, mcr-1	(Clemente et al., 2021)
31.	Pigs and chicken	*E. coli* and *K. pneumoniae*	mcr-1	(Liu et al., 2016b)

Antibiotic resistance is spread from food-producing animals to the environment through the discharge of antibiotics in urine or feces into surface waters and soils, or the use of animal manure as fertilizer to soil or ponds. In primitive economies, untreated animal excrement is used for several reasons. Poultry waste is extensively used in aquaculture to feed fish and shellfish (Zhu et al., 2013). Uncleaned drinking water supplies also expose humans and animals to ABR in the environment. A total of 36% of *E. coli* isolates from Dhaka water supply samples were ABR (Talukdar et al., 2013). Human scavenging and medical waste recycling are both desirable at refuse sites, increasing the danger of ABR exposure (Patwary et al., 2011). Heavy metals and other pollutants in these wastes often co-select for ABR, increasing the release of more ABR genes (Zhu et al., 2013).

Antimicrobial resistance genes are also reported from various environmental samples. As evidenced by the elevated ABR gene contamination of rural Indian river waters during the seasonal pilgrimage of urban citizens to a sacred place on the river, anthropogenic influences on the resistome have been hypothesized from “natural” experiments (Ahammad et al., 2014). Human antibiotic usage and environmental contamination have been linked. In a hospital in India, Diwan et al. (2010) compared the amounts of frequently prescribed antibiotics to the antibiotic concentrations and susceptibilities of *E. coli* in hospital-associated water. A significant correlation was observed with ciprofloxacin being the most common prescribed antibiotic and having the highest concentration in water. ABR gene, blaNDM-1 was found in drinking-water samples and seepage samples from New Delhi (India), according to a study by Walsh et al. Bacteria with blaNDM-1 were found, including *Shigella boydii* and *Vibrio cholerae*, among 11 species in which NDM-1 had not previously been described (Walsh et al., 2011). A similar study was conducted by Shahid et al. wherein they found only CTX-M among the environmental samples and none of the isolates showed NDM-1 (Shahid, 2011; Shahid et al., 2012). Another study from Northeast India reported the presence of TEM in river water samples (Upadhyay and Joshi, 2015). The co-occurrence of mcr-9, KPC, and cfr genes in recreational areas was emphasized in a study (Furlan et al., 2021). Anthropogenic activities have had a significant impact on marine habitats (Furlan et al., 2021). A study in Saudi Arabia reported *E. coli* isolates 70.8% antibiotic resistant, primarily to amoxicillin and ampicillin and these were from human, fresh vegetables, water, and air, and norfloxacin for human isolates (Aabed et al., 2021). A study in Lebanon reported that *E. coli* and *S. aureus* was significantly higher in lettuce samples (42.30%,51.5%) than in parsley (13.8%, 38%). This study demonstrated that lettuce and parsley which are usually consumed raw may contain pathogenic microorganisms and represent a risk for human health (Halablab et al., 2011). Another study from Italy reported the prevalence of ESBL-producing strains from fresh vegetables (83.3%) and 16.7% for AmpC. Among the 20 bacterial isolates from ready to eat salads, 80% were ESBL-producing strains and 20% as MBL-producing strains (Iseppi et al., 2018). Recent years have seen the emergence of the microbiological risk associated with consuming ready-to-eat food, and numerous outbreaks of pathological microorganisms have brought attention to the fact that fresh vegetables are potential carriers of resistant pathogens that cause foodborne illnesses (Iseppi et al., 2018).

The actions of wild creatures, particularly those that migrate, exacerbate antibiotic resistance condition, which can facilitate the transfer of bacterial antibiotic resistance across borders or to remote areas (Hernando-Amado et al., 2019). Twice a year, about 5 billion migrating wild birds travel across continents, aiding in the spread of various illnesses throughout the world. Several pathogenic bacterial species, including *Escherichia coli, Salmonella, Staphylococcus* spp., *Campylobacter*, and *Listeria monocytogenes*, were isolated from wild birds (Elsohaby et al., 2021). There have also been reports of these pathogens being indirectly transmitted to people (Tsiodras et al., 2008).A study in Saudi Arabia reported multidrug-resistant *E. coli* and *Staphylococcus* spp. in 13 (14.4%) and 7 (18.9%) isolates, respectively. The *E. coli* ABR associated genes blaCTX-M, blaTEM, blaSHV, aac(3)-IV, qnrA, and tet(A) were identified in 7 (7.8%), 5 (5.6%), 1 (1.1%), 8 (8.9%), 4 (4.4%), and 6 (6.7%) isolates, respectively. They also stated that in terms of migratory wild bird species, aquatic-associated species had relatively higher levels of bacterial recovery, mixed infection, MDR, and ABR index. Overall, the findings indicated that migrating wild birds near Al-Asfar Lake may serve as a reservoir for ABR bacteria, giving them the ability to play a part in the development, maintenance, and spread of ABR bacteria (Elsohaby et al., 2021). There are also reports of alarming levels of ESBL-producing *E. coli* in wild birds in countries as Spain (74.8%), Netherlands (37.8%), England (27.1%), Sweden (20.7%), Latvia (17.4%) and Portugal (12.7%), and higher than that reported in Portugal (12.7%), Ireland (4.5%), Poland (0.7%), and Denmark (0.0%) (Stedt et al., 2015). In central parts of Chile, the ESBL-positive *E. coli* strains were isolated from fecal flora of Franklin’s gulls and the detection rate was higher than that from local human (Hernandez et al., 2013). Another study in Saudi Arabia reported that MDR was more serious among *Enterobacter* strains isolated from migratory birds than local resident birds (Abo-Amer and Shobrak, 2015). The study in Sweden indicated that a potential of AR transfer between the human population and wild birds exists even in countries with a low level of ABR (Bonnedahl et al., 2010). According to the study carried out along the northeastern coast of the United States, ABR was more widespread in bacteria isolated from seabirds than those isolated from marine mammals (Rose et al., 2009). Due to their large numbers and diverse range of activities, birds, particularly migratory birds, require much more attention when it comes to their responsibilities in mediating the movement of environmental ABR. Effective ABR management should be built on global, ubiquitous strategies (Wu et al., 2018). According to a study by Wheeler et al., increased human-animal population overlap may increase the potential for the genesis of novel diseases in wildlife (2012). In the Galápagos Islands, the degree of microbiological connectivity between humans and wildlife was measured using antibiotic resistance as a genetic marker. In contrast, no resistance was observed at protected beaches on more remote islands. This finding suggests that human contact may be the source of antibiotic-resistant enteric bacteria (Salmonella and E. coli) in Galápagos wildlife (Wheeler et al., 2012)

Apart from determining the prevalence of ABR, there is a growing necessity to comprehend the customs, behaviors, and practices that drive resistance progression and transmission. Rural households frequently share their living quarters with cattle (Stålsby Lundborg et al., 2015). Humans are exposed to resistant enteric bacteria through behaviors related to the slaughter and preparation of food animals. During the killing of poultry, family members frequently congregate to pray. Only 14% of observers in residential settings indicated handwashing with soap after killing a chicken (Shanta et al., 2017). In small-scale animal-food processing facilities, biosecurity precautions are frequently inadequate or nonexistent. Observations within an abattoir in Ethiopia revealed the absence of running water, soap, and disinfectant during slaughter; cleaning blades, washing hands, and disinfecting the abattoir with the same buckets of water (Dulo et al., 2015). Other potential ABR transmission sources include common surface waters where humans bathe, fish, or wash clothes and household items. While grazing and defecating close, animals use the same water for washing and drinking (Finley et al., 2013). Although there is more information regarding antibiotic usage in agriculture, there are likely to be many more unrecorded practices. Antibiotics are infrequently added to unpasteurized milk before it is sold in unsterilized plastic containers, according to anthropological research among Somali pastoralist tribes (Carruth et al., 2017). This is due to the judicious use of easily available, low-cost antibiotics, as well as modern food processing and storage modifications to enhance the shelf life of milk products (Rousham et al., 2018). The evolution of resistance may be sped up by exposing environmental bacteria to antibiotics as well as a significant population of resistant bacteria, which will also increase the distribution and abundance of resistance genes within the resistome, which is crucial for the emergence of clinical resistance, as well as the transfer of antibiotic resistance genes between bacteria. Antibiotic resistance should be considered within the “One Health” concept, which offers a worldwide approach to enhancing interdisciplinary collaboration and communication because people and animals are interconnected through the environment (Finley et al., 2013).

## Health risks (human, animal & environment) from antibiotic usage

ABR characteristics include chronic illnesses, delayed action, and the spread of resistance to other species. The emergence of ABR and ABR healthcare-associated illnesses has resulted in a slew of clinical issues (Samreen et al., 2021). Each year, more than 2.8 million antibiotic-resistant illnesses occur in the United States, resulting in more than 35,000 deaths. When you add in *Clostridioides difficile*, a bacterium that is usually not resistant but can cause severe diarrhea and is linked to antibiotic use, the total number of infections approaches 3 million and mortality of 48,000in the United States (The Centers for Disease Control and Prevention, 2019).

Antibiotic residues from the environment may largely infiltrate the human gastrointestinal tract, which is home to around 800–1000 distinct bacterial species and over 7000 different strains (Jernberg et al., 2010). *Bacteroidetes* and *Firmicutes* dominate and there is a micro-ecological balance between these bacteria and bacteria and the human body over time (Arumugam et al., 2011). When the intestinal microbiota is out of balance due to antibiotic therapy, it can lead to the multiplication of harmful bacteria and opportunistic pathogens, which can lead to illnesses including pseudomembranous colitis, intestinal problems, and colorectal cancer (Damman et al., 2012). The effect of some of the commonly used antibiotics on human/animal health is shown in **Table 4**. Individuals as well as communities are affected by the issue of resistance. According to an assessment of patients treated with antibiotics for bacterial urinary tract and respiratory tract infections, individual resistance to antibiotics can continue up to 12 months following treatment. As a result, situations arise where second-line medications are essential (Costelloe et al., 2010). *Pseudomonas aeruginosa, Acinetobacter baumannii, Clostridium difficile, Burkholderia cepacia*, and *E. coli, Klebsiella pneumoniae*, *Hemophilus influenzae*, *Campylobacter jejuni*, and *Salmonella* spp. are Gram-negative organisms that cause nosocomial (hospital-associated) infections. These pathogens are responsible for a variety of diseases in people and animals (Hawkey, 2015). In 1985, scientists in Arizona linked raw milk intake to a multidrug-resistant Salmonella enterica serovar Typhimurium epidemic that resulted in the death of a 72-year-old woman. Most patient isolates were identical to milk isolates, and plasmid research revealed that they all carried the same resistance plasmid (Tacket et al., 1985). *Salmonella Typhimurium* bacteria with nalidixic acid resistance and lower fluoroquinolone susceptibility caused an outbreak in Denmark in 1998. *Salmonella* strains from patients, pork’s, the swine herds of origin, and the slaughterhouse all had a unique resistance pattern, according to PFGE (Molbak et al., 1999). Nontyphoidal *Salmonella* is a foodborne pathogen linked with human gastroenteritis, is commonly transferred by animals in transit, infecting animal meat products and poultry through carrier animal excrement (Sodagari et al., 2020). Salmonella resistant to fluoroquinolones and cephalosporins is a global public health concern (Dutil et al., 2010).

**Table 4:** Impact of some of the common antibiotics on human/animal health* .

Authors	Source	Antibiotics	Human therapeutic significance	Impact on human/animal health
(McGlinchey et al., 2008)	Sheep, cattle, poultry, piglets, lambs, goats & turkey	Aminoglycosides	Critically important	Renal dysfunction and necrosis, cardiac dysfunction
(Jan, 2017)	Eggs, milk	Amoxicillin	Critically important	Carcinogenic effects
(Olatoye and Ehinmowo, 2010)	Raw milk, beef and chicken, calves	Oxytetracycline	Highly important	Cytotoxicity in the bone marrow of broiler chicken & Carcinogenic effects
(Salama et al., 2011; Guetiya Wadoum et al., 2016)	Beef and chicken.	Tetracycline	Highly important	Alteration of the normal intestinal microflora, nephrotoxicity, carcinogenicity, hepatotoxicity, permanent tooth discoloration, acidosis in proximal and distal renal tubules, skin hyperpigmentation on exposure to sun
(Hornish and Kotarski, 2002)	Poultry, cattle, goat, pigs, sheep and turkey	Cephalosporines	Critically important	Anorexia
(Cheong et al., 2010)	Raw milk	Sulfonamides	Highly important	Allergic reactions and carcinogenic effects
(Nirala et al., 2017)	Raw milk	Sulfadimidine, sulfamethoxazole	Highly important	Allergic reactions and carcinogenic effects
(Er et al., 2013)	Chicken and beef	Quinolones	Critically important	Tendon rupture, hypersensitivity reactions, and phototoxicity on skin

(Samreen et al., 2021).

Fears about the human health hazards of ABR related to environmental antibiotic residues include (1) the possible threat of consumed antibiotic residues modifying the human microbiota and promoting the rise and selection of resistant strains of bacteria, known as human antibiotic resistance (Cho and Blaser, 2012); and (2) the possible danger of generating a selection pressure on the environmental microflora resulting in ABR bacteria and ABR genes reservoirs, also known as environmental antibiotic resistance (Qiao et al., 2018).

## One Health approach to combat ABR

Crucially it is difficult to know how much the aforementioned transmission and selection factors contribute to the current rising prevalence of ABR and rising incidence of drug-resistant infections (Booton et al., 2021). The relationship between antibiotic use and resistance is exceedingly complex and reliant on pre-existing bacterial population patterns. Antibiotic use in humans and animals, environmental contamination from those sources, or antibiotic use within non-animal agriculture are regarded to typically increase the prevalence of ABR (Holmes et al., 2016). Previous research has revealed that no single “silver bullet” method occurs to account for this. Instead, attempting to prevent and decrease the burden of ABR in a One Health system should take a multifaceted (Van Puyvelde et al., 2018), joint approach by focusing on the specifics of antibiotics use, as well as the varieties and prevalence of ABR in each system, while taking into account possible interactions between and within systems (Holmes et al., 2016). A collaborative approach is required at various levels such as international, national, community, hospital, individual, patient, animal, agriculture and environment level.

### Important pillars of support to be considered to combat ABR under one health approach

•Rigorous surveillance using established and new surveillance systems across different sectors in combination or individually is an important containment measure to be followed to combat ABR. The use of surveillance allows for the quick detection and study of drug-resistant bacteria outbreaks as well as the tracking of the spread of resistant microorganisms in specific populations or geographic regions. Findings from surveillance will provide accurate guidance for treatment activities to be performed, guide policy suggestions, and assess the effectiveness of prevention and control measures used to control the infection (Nasir et al., 2015). During surveillance, data is gathered and analyzed in order to identify and follow public health hazards. The epidemiology of the threat and the burden it imposes on the populace should also be disclosed through surveillance. The timely dissemination of data to stakeholders with the purpose of inspiring action targeted at decreasing or mitigating the public health concern under observation is another essential element of surveillance. Antibiotic susceptibility test data on bacteria isolated from clinical samples sent for analysis are collected by microbiology laboratories as part of the surveillance of antimicrobial resistance. These data can be correlated with demographic and clinical information for the patient groups from where the microbes were isolated to get insight into the underlying epidemiology and to make it easier to create effective interventions to reduce the burden of resistance (Johnson, 2015).

•Major issues faced by developing countries include the lack of competent laboratories, poor infrastructure and data management, lack of standard protocols, low surveillance coverage, lack of intersectoral cooperation, and inadequate national, regional and international collaboration. The ability of microbiological laboratories to correctly identify resistant bacteria is crucial for AMR surveillance (Ondoa et al., 2017). Emerging infectious diseases cannot be diagnosed in a timely manner because of a lack of laboratory-based surveillance. For instance, Tuberculosis caused by *Mycobacterium tuberculosis* is an emerging disease in humans and leading cause of deaths in adults worldwide (Alexander et al., 2002). It is essential to set up sentinel surveillance labs that are well-equipped in every province to stop the spread of resistant organisms. It is challenging to compare data between laboratories across the nation since there are no standardized protocols for measuring resistance, which causes heterogeneity in the techniques applied. The one health approach can be fully incorporated into the ABR surveillance if and only if intersectoral interaction exists (Bunduki et al., 2019).

•Core actions to be taken by national and international organizations.

One health supports collaboration among physicians, veterinarians, and others to solve complicated issues like ABR affecting diverse species and infections in changing environments. The American Medical Association, the Centers for Disease Control, and the World Health Organization have all expressed support for the concept (Kahn, 2009). Academic medical institutions have been urged to implement One Health transdisciplinary education and research methodologies (Allen-Scott et al., 2015). At the regional level, One Health education, according to Villanueva-Cabezas et al, should extend beyond medical and veterinary students and begin before postgraduate programs. Transdisciplinary thinking and teamwork are best learned in rich diverse student cohorts, including literature, humanities, and sociology, as well as science, technology, engineering, mathematics, and medicine (Villanueva-Cabezas et al., 2022). This innovative strategy was applied at the University of Melbourne through a sequence of two University breadth courses at the undergraduate level. Undergraduate students are expected to study disciplines outside of their home faculty to gain a broader set of abilities where the breadth of topics are a subset of these areas, aiming to teach students complementary ways of thinking about issues and problems while also questioning their assumptions (Krohn, 2016). The One Health concept has been implemented in various ways at the national and international levels including The Lancet One Health Commission in 2019 which was convened with the aim “*to synthesize the evidence supporting a One Health approach to enhancing health within an environment shared by humans and animals*”. The Commission consists of 26 commissioners and several international researchers from multidisciplinary areas who plan, support and recommend integrated approaches from a One Health concept (Institute of Health and Society, 2019).

To combat ABR, the WHO has created a Global Action Plan centered on One Health, which requires all members throughout the world to prepare national action strategies using the same principles. A better knowledge of ABR is required, which can be accomplished by excellent communication, education, and training. Physicians, vets, agriculturists, entrepreneurs, and regulators are all One Health stakeholders who should be acquainted with the ABR One Health domains. These elements could reduce antibiotic use in humans, animals, and farms while also limiting ABR spread in the environment (McEwen and Collignon, 2018). Queenan et al. have suggested a conceptual model for a One Health approach to ABR surveillance, where it centralizes and unifies antibiotic surveillance for humans and animals, as well as ABR data from animals, humans, food, and the environment (Queenan et al., 2016).

To facilitate the systematic gathering of data internationally, the WHO Global antimicrobial resistance Surveillance System (GLASS) and the Organization for Animal Health (OIE) global database on the use of antibiotics in animals was established. GLASS had enrolled 9 of 11 WHO South East Asia area nations and 6 of 27 WHO Western Pacific Region countries by 2018, however surveillance data reporting is restricted (Global antimicrobial resistance surveillance system (GLASS) report). Successful regional AMR surveillance networks such as Central Asian and Eastern European Surveillance of Antimicrobial Resistance (CAESAR), the European Antimicrobial Resistance Surveillance Network (EARS-Net), and Red Latinoamericana de Vigilancia de la Resistencia a Los Antimicrobianos complement GLASS and OIE’s functions in other parts of the world (ReLAVRA) (Ashley et al., 2018).

Economic constraints, dispute, civil conflict, social upheaval, political unrest, mass migrations, and transborder animal mobility have all had an impact on the onset, regulation, and control of zoonotic diseases such as avian influenza, brucellosis, rabies, Crimean–Congo hemorrhagic fever, Middle Eastern respiratory syndrome, and transboundary animal movement in the WHO Eastern Mediterranean Region (Mahrous et al., 2020). When the Middle East Respiratory Syndrome Corona Virus (MERS-CoV) first appeared in the Middle East in 2012 (Zaki et al., 2012), there was a lot of confusion about its epidemiology and clinical manifestations. When it was discovered that dromedary camels were the virus’s natural reservoir, public health systems across the Arabian Peninsula faced tremendous pressure to prevent the virus’s spread (Sikkema et al., 2019). In the years 2012–2017, Qatar employed a single-health approach to managing the MERS-CoV outbreak. The Qatar National Outbreak Control Taskforce (OCT) was reactivated in November 2012 to handle the MERS outbreak in Qatar using a One Health strategy. The OCT invited the animal health sector to participate. Technical assistance was thereafter sought from the WHO, FAO, CDC, EMC, and PHE. Following that, *via* coordination and collaboration, monitoring and inquiry, epidemiological research, and increased local diagnostic capacity, a comprehensive One Health plan was developed and executed (Farag et al., 2019). Awaidy and Hashami concluded in their article that in Oman, even though the number of zoonotic diseases is less, the risk of developing zoonoses stays high due to globalization, resource development, and climate variability. However, to reduce the socioeconomic and public health impact of new zoonotic illnesses in Oman, the country should adopt a “One Health” approach that considers current regional and global strategies for prevention, control, and subsequent elimination of zoonotic diseases (Awaidy and Al Hashami, 2019).

Since antibiotic resistance is discovered in one of the most essential resources for life – water, The Centers for Disease Control and Prevention (CDC) recently met with international experts from across One Health to discuss this important problem and learn more about the vital work being done to track antibiotic resistance in the water, assess its impact on public health, and act to address this potential threat. This discussion took place as part of the CDC’s AMR Exchange, a new worldwide webinar series that debuted in May 2021 to engage a wide range of partners, practitioners, and policymakers in the fight against antibiotic resistance (The Centers for Disease Control and Prevention, 2022).

At the European level, The European Centre for Disease Prevention and Control (ECDC) coordinates EARS-Net, which monitors ABR in invasive bacteria isolated from blood and cerebrospinal fluid in hospitalized patients [European Centre for Disease Prevention and Control (ECDC)], as well as the European Food- and Waterborne Diseases and Zoonoses Network (FWD-Net), which monitors ABR in human *Salmonella* and *Campylobacter* infections [European Centre for Disease Prevention and Control (ECDC)]. According to Directive 2003/99/EC (European Commission) and Decision 2013/652/EU (European Commission), the European Food Safety Authority (EFSA) coordinates mandatory active monitoring of AMR in zoonotic bacteria (*Salmonella and Campylobacter*) and indicator bacteria (*Escherichia coli*) from healthy food-producing animals (cattle, poultry, pigs) and their food (European Food Safety Authority (EFSA) et al.). The United Kingdom (UK) government released the ‘UK five-year AMR strategy (2013 to 2018) in 2013, outlining efforts to slow the growth and progression of AMR using a One Health strategy. The UK government established the Antimicrobial Resistance Review, which published its final report in 2016 and included endorsements to combat AMR, including steps on infection prevention and control practices, as well as a decrease in antibiotic use in animals and humans. The European Commission (EC) released its “European One Health Action Plan against AMR” in 2017 (Veterinary Medicines Directorate, 2019). The EU summary report on AMR in zoonotic and indicator bacteria from humans, animals, and food is published once a year by ECDC and EFSA.(European Food Safety Authority (EFSA) et al.) These coordinated systems demonstrate substantial efforts at the European level to collect useful AMR data from a public health standpoint (Mader et al., 2021).

In Canada, the need for new understanding regarding the effectiveness and economic efficiency of integrated AMR surveillance systems has been highlighted, where the Canadian Integrated Program for Antimicrobial Resistance Surveillance (CIPARS) has been in place since 2002. CIPARS led by the Public Health Agency of Canada (PHAC) collects, integrates, analyzes, and communicates trends in antimicrobial use (AMU) and AMR in specific bacteria isolated from animals, humans, and animal-derived food products across Canada (Public Health Agency of Canada). The CIPARS’ goals are to provide a holistic approach to monitoring AMU and AMR patterns in humans and animals, to make it easier to evaluate the public health impact of AMU in humans and agriculture, and to make precise estimates with information from other nations of comparable surveillance systems (Aenishaenslin et al., 2021).

The newly released Global Health Security Agenda (GHSA) 2024 Framework and the GHSA goals were shaped in part by United States Agency for International Development (USAID). Since 2015, the Agency has worked with over 17 GHSA nations to develop zoonotic disease and One Health capacity by bringing together expertise in animal, human, and environmental health. The Agency assists focus nations in preventing, detecting, and responding to infectious disease threats that threaten global health security through strategic capacity-building operations and utilizing current US Government and partner investments. Indonesia sponsored the 5^th^ Global Health Security Agenda (GHSA) Ministerial Meeting in November 2018. The forum emphasized the importance of maintaining global health security in the face of infectious disease threats, whether natural, unintentional, or deliberate. All 64 GHSA member countries agreed to extend the organization’s original five-year mandate to 2024 (The United States Agency for International Development).

AMR claimed the lives of 255 000 people in Sub-Saharan Africa in 2019, with more than half of them being children under the age of five. As a result, Ethiopia is stepping up its energies to effectively combat ABR using a One Health approach (The Food and Agriculture Organization of the United Nations, 2022). The AMR Multi-Partner Trust Fund (AMR MPTF) initiative in Ethiopia has been instrumental in beginning and developing scientific guidelines for safe and high-quality animal source food production and AMR control. The Ministry of Agriculture (MOA) took the lead and developed a One Health interdisciplinary team that included the MOA, Ethiopian Food and Drug Authority, National Sanitary and Phytosanitary Steering Committee, Veterinary Drugs and Animal Feed Administration and Control Authority, Ethiopian Public Health Institute, Animal products, food safety lab of the Veterinary Drug and Feed Quality Testing Center, Ethiopia Standards Agency, and others (The Food and Agriculture Organization of the United Nations, 2022).

The Australian government issued the 2020 Strategy to tackle AMR in March 2020. The One Health Master Action Plan (OHMAP) offers advice on how to put Australia’s National Antimicrobial Resistance Strategy – 2020 and Beyond into action (the 2020 Strategy). The 2020 Strategy is divided into seven goals: antimicrobial resistance programs must have clear governance; prevention and control of the spread of resistance and infections; increased participation in the campaign against resistance; proper stewardship practices; coordinated monitoring and response to resistance; a comprehensive research agenda involving all sectors; and increase international collaboration and alliances (amr.gov.au). The Ministry of Primary Industries and the Ministry of Health in New Zealand are also working together to reduce the effect on the plant, animal, and human health. The Antimicrobial Resistance Action Plan for New Zealand was announced at the 70th World Health Assembly. This action plan was created in collaboration with partners from New Zealand’s human, animal, and agricultural sectors to address areas where the action is needed. It has five main goals, all of which are in line with the World Health Organization’s Global Action Plan (Ministry of Health and Ministry for Primary Industries, 2017).

The Joint Programming Initiative on AMR, White House, and WHO recommendations for a global One Health strategy will ideally impact the national systems committed to being part of the universal system. Building capacity at the local and national levels remains a serious problem. Although technological advances may contain some big data solutions, implementing high-tech systems in rural, impoverished areas may be problematic. However, emerging countries may have the largest impact on the development of ABR. Global funding is required to improve the control of hospital infections, control antimicrobial usage in food-producing animals, and limit the sale of medicines without the need for prescription and counterfeit drugs (Queenan et al., 2016). It is proposed to take a broadened approach to evidence gathering, surveillance, data analysis, intervention design, and assessment. Considering the various challenges and losses that will be faced will be recovered by demonstrable financial efficiency and improved human and animal health outcomes. Furthermore, a One Health approach to ABR surveillance is bolstered when the less tangible benefits to society are considered (Queenan et al., 2016).

## Conclusion

The dissemination of ABR bacteria and ABR genes globally is a public health concern. A multi-sectoral and integrated One Health approach to addressing ABR is possible. The pillars of support including rigorous ABR surveillance among different sectors individually and in combination, and at national and international level, overcoming laboratory resource challenges, and core plan and action execution should be strictly implemented to combat and contain ABR under one health. The global strategic plan will need to take a consistent, coordinated, multidisciplinary, and interdisciplinary approach, as well as global coordination and continuing data exchange across all colleagues involved, in ABR surveillance. Several national and international organizations have included, advocated, and launched a One Health concept in their antimicrobial resistance action plans. The global assessment of the One Health approach and the FAO-OIE-WHO vow would help ABR prevention through awareness programs, education about antibiotic use practice, political commitment, and antimicrobial stewardship. Implementing One Health could help to avoid the emergence and dissemination of ABR while also maintaining a steady supply of effective antibiotics and a healthier One World.

## Author contributions

NA conceptualization and drafted the first version of the manuscript. RJ drafted the first version of the manuscript. MS conceptualization reviewed the draft and edited it substantially. All authors approved the final version of the manuscript. All authors contributed to the article and approved the submitted version.
